# Oral squamous cell cancer: early detection and the role of alcohol and smoking

**DOI:** 10.1186/1758-3284-3-2

**Published:** 2011-01-06

**Authors:** Anna G Zygogianni, George Kyrgias, Petros Karakitsos, Amanta Psyrri, John Kouvaris, Nikolaos Kelekis, Vassilis Kouloulias

**Affiliations:** 1 Kapodistrian University of Athens, Medical School, Radiation Oncology; Greece; 2 Thessalia University, Medical School, Radiotherapy Dpt, Larissa; Greece; 3 Department of Cytopathology, ATTIKON, University Hospital, Athens, Greece; 4 Attikon University Hospital, Medical Oncology Unit, XAIDARI, Greece

## Abstract

**Objective:**

Oral squamous cell carcinoma has a remarkable incidence worldwide and a fairly onerous prognosis, encouraging further research on factors that might modify disease outcome.

**Data sources:**

A web-based search for all types of articles published was initiated using Medline/Pub Med, with the key words such as oral cancer, alcohol consumption, genetic polymorphisms, tobacco smoking and prevention. The search was restricted to articles published in English, with no publication date restriction (last update 2010).

**Review Methods:**

In this review article, we approach the factors for a cytologic diagnosis during OSCC development and the markers used in modern diagnostic technologies as well. We also reviewed available studies of the combined effects of alcohol drinking and genetic polymorphisms on alcohol-related cancer risk.

**Results:**

The interaction of smoking and alcohol significantly increases the risk for aero-digestive cancers. The interaction between smoking and alcohol consumption seems to be responsible for a significant amount of disease.

**Conclusion:**

Published scientific data show promising pathways for the future development of more effective prognosis. There is a clear need for new prognostic indicators, which could be used in diagnostics and, therefore a better selection of the most effective treatment can be achieved.

## Introduction

In the past, squamous cell carcinoma of the oral cavity (OSCC) was primarily found in elderly men with the risk factors being tobacco and excessive alcohol use. However, some studies have shown an increased incidence of OSCC among young patients under 40 years of age [1]. According to Llewellyn et al and Manuel S et al recent case-control studies there are controversial results concerning the possible differences in the etiology and biological nature of OSSC between young and elderly patient groups. The two studies have indicated that OSCC is a similar disease in the age groups under and over 40 years [2,3].

Primary OSCC is treated by surgery with or without neck dissection, or by combined surgery and radiotherapy. Despite the radical nature of the treatment, recurrences are common [4]. There is a clear need for new prognostic indicators, which could be used in diagnostics and, consequently, in the selection of the most effective treatment method [5].

## Methods

A web-based search for all types of articles published was initiated using Medline/Pub Med, with key words such as oral cancer, alcohol consumption, genetic polymorphisms, tobacco smoking and prevention. The search was subsequently refined. The sites of specialized scientific journals in the areas of oral and maxillofacial surgery, oral medicine, and oncology were also used. We give an overview of published studies on the combined effects of alcohol drinking, smoking and polymorphisms in genes for alcohol dehydrogenase (ADH), aldehyde dehydrogenase (ALDH), cytochrome P450 2E1, and methylene-tetrahydrofolate reductase on the risk of alcohol-related cancer. Other available data are insufficient or inconclusive and they highlight the need for additional studies. The search was restricted to articles published in English, with no publication date restriction (last update 2010).

## Review of the literature

### Patient related factors

There are no prognostic differences between males and females, although some authors have reported lower survival rates in females, attributed to delay in seeking medical care and lower acceptance of treatment [6,7]. The correlation of prognosis with age seems controversial, and some authors show no relationship between them, whereas others demonstrate worse prognosis in older patients [8]. Another possible theory is that patients with more hostile tumours develop symptoms earlier, so they seek medical attention sooner; nevertheless, these patients still have to face a more grievous outcome, because these malignancies display a more aggressive biologic behaviour [9].

### Genetics

Fialka F et al in a microarray-based gene-expression analysis found 601 genes to be significantly regulated in cancer tissue compared to adjacent intra-individual mucosa controls, and 25 genes with differences in their regulation comparing samples from early-stage cancer with the ones from advanced disease. Genes FMO2, CPA6, TNC, and SIAT1 were significantly up regulated in early disease stages, and LGI1 gene expression was significantly enhanced in normal adjacent mucosa of patients with early-stage disease without showing a differential expression in carcinoma biopsies [10].

Chiang WF et al have established the amplification, mutation and expression of one gene - epidermal growth factor receptor (EGFR) - in areca-associated oral squamous cell carcinoma, showed amplifications of EGFR in 33% of cases. Significant increases in EGFR copy number and EGFR immunoreactivity were found in OSCC compared with matched adjacent oral mucosa, suggesting that genomic amplification could be a genetic basis underlying activation of the EGFR pathway in areca-associated OSCC [11].

A study of Hatagima A et al of genetic polymorphisms of the carcinogen-metabolising enzyme Glutathione-S-transferase at GSTM1, GSTT1, and GSTP1 gene loci on OSCC susceptibility among Brazilians did not support the hypothesis of an increased risk of GSTP1 G/G, GSTM1 or GSTT1 null genotypes for developing OSCC: rather the GSTM1 A/B genotype emerged as a protective factor [12].

The research of Serefoglou Z et al has indicated functional polymorphisms affecting gene expression of interleukins IL-4, -6, -8, and -10 as well as tumour necrosis factor-alpha (TNF-α), are strongly associated with an increased risk for OSCC [13]. Smokeless tobacco has been shown to induce TNF-α, which, along with its receptors, is over-expressed in OSCC. Single nucleotide polymorphisms (SNPs) in TNF-α and TNF receptor genes may affect their expression and may be a potential determinant of susceptibility to tobacco-related OSCC. TNF-α -308 G allele was significantly lower, whereas A allele was significantly higher in OSCC patients compared with controls. Both TNFR1 -609 TT and TNFR2 1690 CT genotypes were significantly lower in OSCC patients compared with controls. It seems that TNF-α -308 G/A may be related to susceptibility, whereas -609 TT TNFR1 and 1690 C/T TNFR2 SNPs may be protective to tobacco-related OSCC [14].

Lin YC et al focused on polymorphisms of COX-2 -765 G > C. The frequency of COX-2 -765 G/G genotype was significantly higher in healthy controls. COX-2 -765 C allele vs. -765 G/G genotype was a protective factor against OSCC development, but was a risk factor for malignant potential of oral potentially malignant disorders. Polymorphisms of p53 codon 72 were not associated with OSCC development and malignant potential of oral potentially malignant disorders [15].

Functional DNA repair genes are essential for the protection against carcinogenesis. Study of the DNA repair gene ERCC6, produced results suggesting that the heterozygous and homozygous A allele of the ERCC6 codon 399 may be associated with the development of OSCC. Those who had homozygous A/A or heterozygous A/G at ERCC6 codon 399 showed increased risk of OSCC compared to those with G/G. As for ERCC6 codon 1097 or 1413, there was no difference in distribution between the OSCC and control groups [16].

Study of the DNA double-strand break repair gene XRCC4, found a significant different distribution in the frequency of the XRCC4 intron 3 genotype, but not the XRCC4 G-1394T or intron 7 genotypes, between OSCC and control groups. Chiu CF et al observed that those who had heterozygous del/ins at XRCC4 intron 3 showed increased risk of OSCC compared the ones with ins/ins. As for XRCC4 G-1394T or intron 7 polymorphisms, there was no difference in the distribution between OSCC and control groups. There were significant gene-environment interactions between XRCC4 intron 3 genotype with smoking and with betel quid chewing, but not with alcoholism. In smoker and betel quid chewer groups, the XRCC4 intron 3 del variants exhibited higher risks than the ins genotype, respectively. These results suggest that the XRCC4 intron 3 del genotype may be associated with OSCC [17]. Individuals who carried at least one C allele (T/C or C/C) of the DNA repair gene Ku70 promoter T-991C had an increased risk of developing OSCC compared to those who carried the T/T wild-type genotype but neither the Ku70 promoter C-57G, promoter A-31G or intron 3, were connected to OSCC susceptibility [18].

Coutinho-Camillo CM et al showed the involvement of th Bcl-2 family proteins in OSCC tumorigenesis and suggests that the expression of apoptotic molecules might be used as a prognostic indicator for OSCC. Two hundred and twenty nine cases of OSCC, arranged in a tissue microarray, were immunohistochemicallyanalysed [19].

Recently Chaudhary A et al focused in matrix metalloproteinases(MMPs), enzymes that degrade all the components of extra cellular matrix and collagen. They studied 362 patients with oral submucous fibrosis (OSMF)and head and neck lesions and they concluded that the expression of MMP-3 genotype associated with 5A alleles may have an important role in the susceptibility of the patients to develop OSMF and head and neck squamous cell carcinoma [20].

The effectiveness of tight junction proteins (claudins 1, 4, 5, 7 and occludin) and cancer associated fibroblasts (CAFs) as prognostic markers in OTSCC and as markers of malignancy in ameloblastomas was studied by Bello I et al. Abundance of CAFs and Claudin 7 derangement was found to be associated with poor disease-specific survival in oral (mobile) tongue cancer. Appearance of CAFs within the epithelial islands of ameloblastoma was found to be a marker of malignancy in the tumor. The prognostic predictability of CAF density, Ki-67 (cell proliferation marker), maspin (tumor suppressor marker) and tumor DNA content (tumor ploidy using image cytometry) in tongue cancers was also tested. CAF density was the only marker strongly predictive of prognosis. In ameloblastomas, α-SMA (for CAFs), Ki-67, epithelial membrane antigen (EMA) and DNA content (using image and flow cytometry) were assessed as markers of ameloblastic carcinoma. Only α-SMA was able to predict ameloblastic carcinoma when it was found in the epithelial islands. In conclusion, staining for α-SMA and claudin 7 seems to be beneficial for prognostication in tongue cancer, while α-SMA staining may be beneficial in differentiating ameloblastoma from ameloblastic carcinoma [21].

### Alcohol consumption

Several researchers [22,23] have reviewed the effects of alcohol-drinking on cancer risk. Ethanol in alcoholic beverages has been classified as carcinogenic to human beings. A causal link has been established between alcohol consumption and cancer of the upper aero digestive tract (i.e., of the oral cavity, pharynx).

Ethanol is absorbed by the small intestine and later metabolised, mainly in the liver. Alcohol dehydrogenases (ADH) are cytosolic, dimeric, zinc-containing NAD-dependent enzymes that oxidise ethanol into acetaldehyde. When alcohol consumption is high, the cytochrome P450 2E1 (CYP2E1, a member of the cytochrome P450 superfamily) can also catalyse ethanol into acetaldehyde while producing reactive oxygen species (ROS) [24]. Subsequently, acetaldehyde is converted into acetate by aldehyde dehydrogenases (ALDH). Although there are multiple forms of ALDH in the liver, the enzyme encoded by ALDH2 on chromosome 12 has a very low Michaelis constant for acetaldehyde (about 1 μmol/L) and is thought to oxidise most of the acetaldehyde generated during alcohol metabolism.

Asakage T et al and Hiraki A et al with the observation [25,26] in Asian populations found a significantly higher risk of cancer of the upper aerodigestive tract, oral cavity or oropharynx, and hypopharynx in moderate or heavy drinkers carrying the ADH1B*1/*1 genotype. In particular, a gene-environment interaction between ADH1B polymorphism and alcohol-drinking was significant (p = 0·035): risk of cancer of the upper aerodigestive tract in heavy drinkers with a ADH1B*1/*1 genotype was higher than for heavy drinkers carrying the ADH1B*2 allele. A recent multicentre study [27] done in European countries by Hashibe M et al found a protective effect of the ADH1B*2 allele in drinkers compared with ADH1B*1/*1. Significantly reduced odds ratios (OR) of 0·57 (95% CI 0·36-0·91) and 0·36 (0·17-0·77) were recorded in never or moderate and in heavy drinkers, respectively, who carried the ADH1B*2 allele. These results are in agreement with the conclusions of a pooled analysis [28] of three multicentre case-control studies (n = 3876) of ADH, alcohol-drinking, and risk of cancer of the upper aerodigestive tract: compared with ADH1B*1/*1 never-drinkers, overall OR for moderate drinkers carrying the *2 allele was 0·65 (0·50-0·85) and for heavy drinkers carrying this allele was 0·42 (0·31-0·56).

Overall, the results obtained for ADH1B polymorphisms do not concord with the so-called acetaldehyde hypothesis that the ADH1B*1 allele (which encodes a less-active enzyme, leading to lower acetaldehyde exposure) should decrease the risk of cancer in drinkers. By contrast, a decreased risk of cancer of the upper aerodigestive tract was recorded in drinkers who carried the ADH1B*2 allele that codes for the more-active enzyme. The increased risk for ADH1B*1/*1 homozygotes might result from an absence of alcohol flushing, enhancing vulnerability to drinking and lifetime exposure to acetaldehyde, increasing the potential for so-called binge-drinking, and longer exposure of the mucosa to ethanol [29].

In the only available study by Risch A et al, concerning the risk of laryngeal cancer, no significant association was found for ADH1B genotype and alcohol consumption in people of white ethnic origin [30].

Regarding three studies [27,31,32] focusing on risk of head and neck cancer in whites and Africans, two [27,31] showed a significant modification of risk with alcohol consumption. Peters and colleagues, regarded as significant the interaction between alcohol consumption and ADH1C genotype [31]. Odds ratio for cancer of the upper aerodigestive tract in heavy drinkers with ADH1C*2/*2 genotype was 7·1 (2·3-22·0) compared with 2·3 (1·4-3·8) for ADH1C homozygous wildtype (ie, *1) or heterozygous individuals. By contrast, a significantly increased risk was noted for European moderate drinkers with ADH1C*1/*1 genotype compared with ADH1C*2 allele carriers [27].

The study by Zavras et al showed an increased risk of oral cancer for ADH1C*1/*2 heavy drinkers of white ethnic origin [33]. For oral, oropharyngeal, and hypopharynx cancers, the two [25,34] studies by Asakage T et al and Bouchardy C et al that we identified were inconsistent: in one study[34], the effects of ADH1C*1/*1 genotype and lifetime alcohol consumption in white individuals were associated with an increased risk; in another study [25], the risk of oral, oropharyngeal, or hypopharynx cancer were greater for Asians who were moderate to heavy drinkers and of genotype ADH1C*1/*2 or ADH1C*2/*2 than for Asians who were moderate to heavy drinkers with ADH1C*1/*1 genotype.

The studies by Asakage T etal and Hiraki A et al [25,26] of Asian populations and risk of cancer of the upper aerodigestive tract, oral cavity, or oropharynx found a significant interaction between ALDH2*1/*2 genotype and heavy (p = 0·013) or moderate to heavy alcohol intake (p = 0·002). By contrast, another study [35] found no significant modification of oral cavity cancer risk with alcohol consumption and ALDH2 polymorphism. In Europeans, in whom the ALDH2 mutant allele at residue 487 is almost absent, a large multicentre study [27] has shown a significantly increased risk of cancer of the upper aerodigestive tract in individuals who were heterozygous or homozygous for any ALDH2 variants at residues +84, +348, and -241 and who drank moderately or heavily; a significant interaction was noted between alcohol consumption and ALDH2 +348 and -241 polymorphisms [27].

For cancer of the upper aerodigestive tract, all studies [25-27] identified showed increased risk in Asians who were moderate or heavy drinkers and carriers of the ALDH2*2 allele. These results are consistent with the expected functional effect of the *2 variant: that reduced ALDH2 activity decreases the elimination of acetaldehyde. Moreover, individuals with ALDH2 deficiency have increased acetaldehyde levels in serum and saliva than do those with ALDH2*1/*1 genotype, and increased frequency of acetaldehyde adducts, sister chromatid exchanges, and micronuclei have been observed in Asian drinkers with ALDH2*1/*2 genotype compared with individuals with ALDH2*1/*1 genotype [24].

Two studies [34,35] have assessed the effect of CYP2E1 polymorphisms and alcohol consumption. Bouchardy and colleagues[34] showed that in white populations, the highest risks of oral cavity or pharyngeal cancer were recorded for the heaviest drinkers, with a significant 7·2-times increased risk for carriers of CYP2E1 c2 and a significant 2·5-times increased risk for those of CYP2E1 c1/c1 genotype compared with moderate drinkers with *c1/c1 *genotype. However, the small number of carriers of CYP2E1 variant alleles has hindered the interaction analysis [34].

Various studies[36-38] have assessed the relation between MTHFR polymorphism at residue 677, alcohol consumption, and cancer risk.

The study by Suzuki T et al [36] have shown that Asian heavy drinkers with MTHFR TT genotype had a significantly decreased risk of head and neck cancer compared with CT and CC genotypes. Significant interactions between heavy drinking and residue 677 TT genotype were observed for head and neck cancer (p = 0·04) [36].

Another polymorphism has been related to modified MTHFR activity [37]. A substitution of A to C at nucleotide 1298 results in an aminoacid residue change from glutamate to alanine. This polymorphism is associated with reduced enzyme activity, but to a lesser degree than is the MTHFR variant at residue 677. Individuals with the CC genotype at residue 1298 have 60% of the enzyme activity of those with the AA genotype.

Capaccio and co-workers have shown that moderate drinkers of white ethnic origin who are either double heterozygous or double homozygous for MTHFR mutations at residues 677 and 1298 had a higher risk of glottic, supraglottic, and oropharyngeal cancer [38].

### Alcohol and Cigarette smoking

It has long been recognized that there is a strong association between heavy alcohol use and cigarette smoking. Approximately 80% of alcohol dependent patients are reported to smoke cigarettes. [39]. In addition, nicotine dependence appears more severe in smokers with a history of alcohol dependence [40].

The concomitant use of tobacco and alcohol contributes to an increased incidence of several malignancies, especially head and neck cancers. Men who both smoke and drink are nearly 38 times more likely to develop head and neck cancers than men who do neither [41].

Talamini et al. [42] observed a similar multiplicative risk for laryngeal cancer with combined alcohol and smoking exposure in European subjects. In addition, prolonged alcohol consumption and smoking exposure augments the risk for a second primary tumor in patients with a previous upper aerodigestive tract tumor [43].

Mutations in the p53 gene were present more often in tumors from alcohol drinkers who smoked cigarettes (76% of the 105 patients studied) than in nondrinkers who smoked cigarettes (42% of the patients) or in nondrinkers who did not smoke (14% of patients). A role for p53 mutations in aerodigestive cancers in general has been suggested by others [44,45].

Wallstrom P et al have observed that the high alcohol consumption and smoking in combination with GSTM1 null genotype is associated with high titers of plasma autoantibodies against the oxidized DNA base derivative 5-hydroxymethyl-2'-deoxyuridine that is used as a potential biomarker of cancer risk and oxidative stress [46].

The cellular mechanisms impacted by combined smoking and alcohol exposure are poorly understood, but molecular epidemiology approaches are providing insights regarding the importance of effects on oxidant/antioxidant pathways and on metabolic pathways involving the cytochrome P450 system.

Cytochromes P450 (CYP) are enzymes (mixed function oxidases) that are predominantly expressed in the liver and were recognized long ago as central in metabolizing drugs, endogenous compounds, and environmental and dietary substances in humans. Specific CYP enzyme activities are influenced by both ethanol and smoking [47-49].

Miksys et al. [50] have reported that another member of the cytochrome P450 family, CYP2B6, is increased in various regions of the brains of smokers and alcoholics with the potential role for the CC genotype contributing to the highest levels of CYP2B6 protein.

Rodriquez M. et al observed that a statistically significant relationship between smoking and loss of Methylguanine-DNA-methyltransferase (MGMT) protein expression. Loss of MGMT expression could be considered an early event in oral carcinogenesis with possible prognostic implications [51].

Znaor A. et al conducted a case control study in Chennai and Trivandrum, South India and they observed a significant dose response on relation with the duration and the amount of consumption of the smoking, chewing and alcohol with the development of the oral and pharyngeal carcinoma [41].

Nicotine and alcohol interactions within both the developing and adult central nervous system (CNS) have been the subject of much investigation and are reviewed elsewhere [52,53]. Dopamine neurotransmission, particularly in the nucleus accumbens of the mesocorticolimbic system, is central to mechanisms regulating CNS effects of both nicotine and alcohol. Each substance works through different proteins and receptors (classically, nicotinic acetylcholine receptors or nAChRs for nicotine and *N*-methyl-D-aspartate or NMDA and g-aminobutyric acid or GABA receptors for alcohol). However, it is clear that ethanol also influences nAChR activation and that nicotine can mediate dopamine-activating properties of alcohol [53,54]. In addition, there is also a role for another important neurotransmitter, serotonin, in the interactions between nicotine and alcohol within the CNS. It is anticipated that insights gained from genetic studies will further enhance our understanding of how smoking and alcohol interact to influence CNS activity [53-55].

### Viruses

A hospital-based case-control study by Pintos J et al in Canada provided further evidence supporting a causal association between human papillomavirus (HPV) infection and tonsil-related cancers. Cases consisted of newly diagnosed patients with squamous cell carcinoma of the oropharynx and OSCC. Controls were frequency matched to cases on gender, age, and hospital. Oral exfoliated cells were tested for HPV DNA by the PGMY09/11 polymerase chain reaction. Serum antibodies against HPV 16, 18, and 31 viral capsids were detected using immunoassay. HPV DNA was detected in 19% of total cases, and 5% of controls but in 43% of tonsil-related cancers (palatine tonsil and base of tongue) [56].

An interesting anecdotal report showed a couple diagnosed synchronously with squamous cell carcinoma of the head and neck where the tumours were positive for HPV16 by PCR and both viral genomes were genetically identical and closely related to HPV16R - the revised European prototype. These tumours appeared to represent transmission between the couple [57].

## Modern methods of oral cytology for OSCC

### Cytomorphometry

The OralCDx BrushTest is a computer-assisted method for the analysis of cellular samples collected using the brush biopsy. Using a neural network-based image processing system, this method can analyze digitalized microscopic images of collected cells to detect oral precancerous and cancerous cells. OralCDx seems to have a sensitivity and specificity of > 90% [58].

### DNA image cytometry

**Figure 1:**
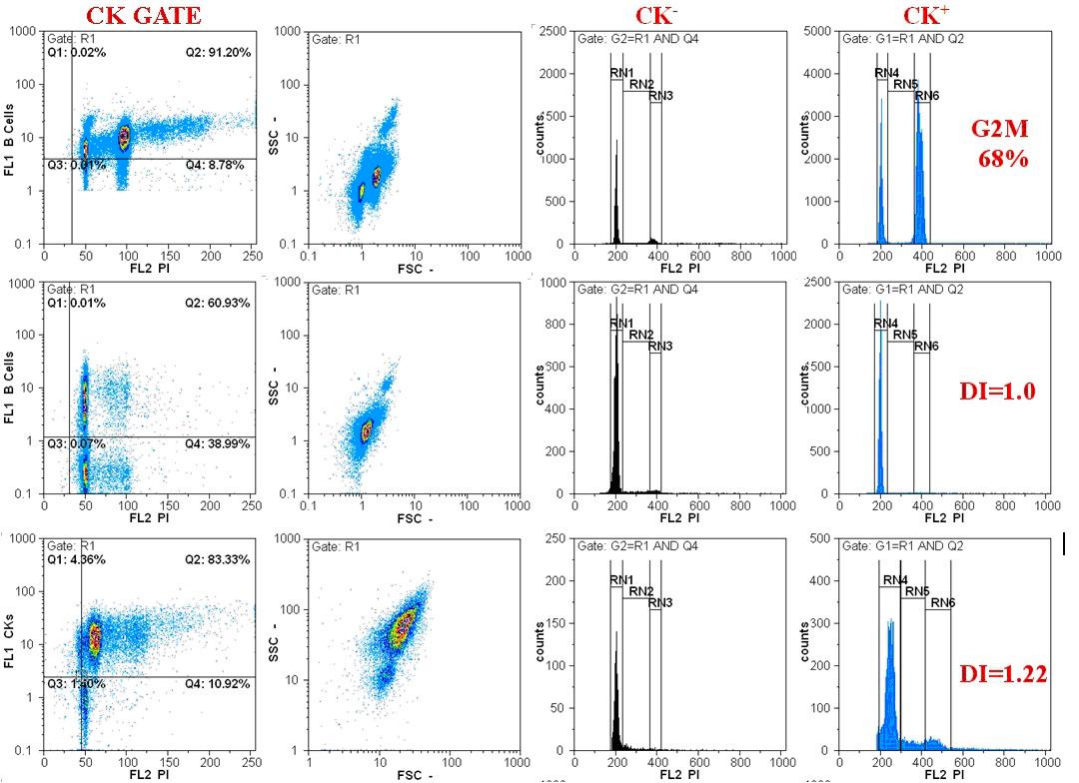
**Flow cytometric analysis of cell cycle using a cocktail of FITC labeled cytokeratins (CK) and propidium iodine (PI)**. Cell populations are gated according to their CK positivity. DNA index (DI) and cell cycle phases (G0/G1, S, G2M) are analyzed for both CK positive and CK negative cells (cytopathology department of ATTIKON university hospital).

DNA cytometry (figure 1.) is a method to measure DNA ploidy. By comparing Feulgen dye-stained cytologic samples with normal epithelial cells, the malignancy of oral mucosal cells can be determined.

Sudbo et al.[59] analyzed archival material and reported that the nuclear DNA content of oral leukoplakia cells can be used to predict the risk of oral epithelial dysplasia up to 5 years before a histologic diagnosis is possible. In addition, a current study reported that promoter hypermethylation was associated with head and neck squamous cell carcinoma[60].

Using a DNA cytometric method, an increase in sensitivity and specificity of the oral brush biopsy to 100% for the early diagnosis of oral cancer was reported [61].

### Optical diagnosis

**Figure 2:**
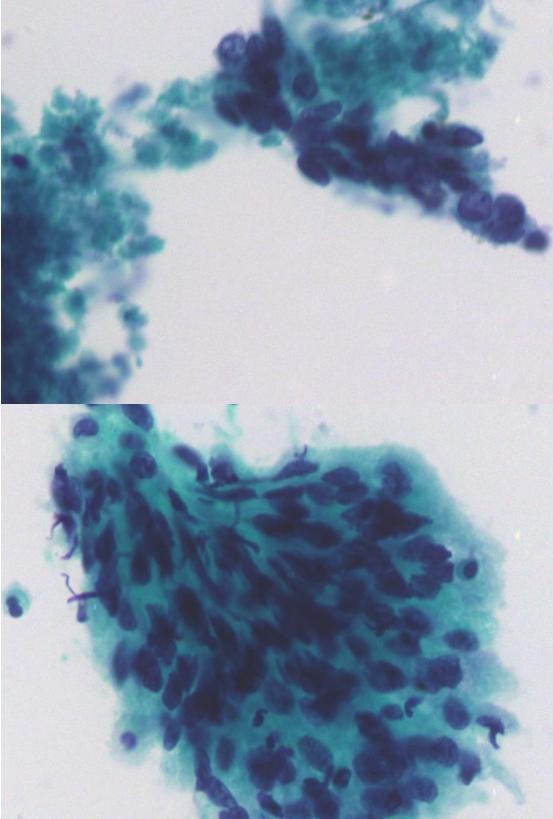
**Poorly differentiated squamous cell carcinoma. ThinPrep^® ^smear of head and neck fine needle aspiration, pap stained ×400**.

An optical diagnosis was proven to be reliable. This was an invasive technique for the detection of fluorescence in tissues that arose from a photosensitizer. The use of autofluorescence to detect malignant lesions emanated from photodynamic therapy, a technique for cancer treatment (figure 2). Autofluorescence describes the biologic characteristic of tissues possessing endogenous fluorophores such as flavin, tryptophan, elastin and collagen, which become fluorescent when exposed to certain wavelengths of light. The presence of disease can change not only the concentrations of these fluorophores but the light-scattering and absorption properties of the tissue too, owing to changes in blood concentration, nuclear size distribution, collagen content and epithelial thickness. The tissue is usually illuminated with a light source, mostly in the near-ultraviolet to green range of the spectrum.

Harris et al.[62] noted endogenous autofluorescence at around 630 nm in tumors of the oral mucosa. However, autofluorescence of normal tissues was also observed. Porphyrin, which may rapidly accumulated in tumors compared with the accumulation of the surrounding normal tissue, it is the most commonly used target in autofluorescence-detecting oncology. Moreover, porphyrin-based tumor detection might only be useful for diagnosing advanced tumors because of its high false-positive potential.

### Photodynamic diagnosis

Photodynamic diagnosis of an oral carcinoma and precursor lesions mainly use 5-aminolevulinic acid (5-ALA), which is a precursor of the fluorescent photosensitizer and protoporphyrin IX (PpIX) which can be administered systemically or applied topically to the oral mucosa and facial skin. Excessive accumulation of 5-ALA-induced PpIX results in the accumulation of intracellular porphyrins, especially PpIX, which increases the tissue fluorescence.

Sharwani et al. [63] used 5-ALA in the form of a mouth rinse to test 71 patients with clinical suspicion of leukoplakia to identify dysplasia or carcinoma *in situ *and they obtained a sensitivity of 83-90% and a specificity of 79-89%.

Chang et al [64] reported that a photodynamic diagnosis, photofrin, was used topically to detect early oral cancer with a sensitivity of 92.45% in a macroscopic study and 93.75% in a microscopic study. The epidermal growth factor receptor (EGFR) is overexpressed in oral cancer.

Soukos et al. [65] used an anti-EGFR monoclonal antibody coupled to the fluorescent dye, *N*, *N*_-di-carboxypentyl-indodicarbocyanine-5,5_- disulfonic acid, to detect a tumor-associated antigen of the EGFR. This method using a specific antibody coupled with a fluorescent dye improved the detection accuracy to specificities of 95.65% in the macroscopic study and 97.50% in the microscopic study.

In addition, 5-ALA-induced PpIX accumulation might lead to selective killing of macrophages at the site of photodynamic treatment in OSCC56 and downregulate the invasion.[66] Recent studies demonstrated improved sensitivity and specificity, and the potential for the immunophotodiagnosis of OSCC. (Table 1.)

**Table 1:** Modern methods of oral cytology for oral squamous cell carcinoma

Classification	Analytic method	Markers
Cytomorphometry	Conventional cytologic diagnosis	Cell morphology
	Computer-assisted image	Cell morphology
	analysis (OralCDx)	

DNA analysis	DNA image cytometry	DNA ploidy, DNA methylation

Optical diagnosis	Autofluorescence	Flavin, tryptophan, elastin,

		collagen (endogenous fluorophores)
	Photodynamic diagnosis	5-Aminolevulinic acid
		Photofrin
	Immunophotodiagnosis antibody targeting	EGFR

An immunocytochemical analysis of oral brush biopsies can recognize atypical cells by detecting altered protein expressions by tumor cells. During the malignant transformation of cells, the structure or expression level of some proteins in the extracellular matrix (ECM) may be altered. The transformation of squamous cell carcinomas with invasiveness and metastatic potential is associated with a poor survival rate. The cell surface molecules involved in cell migration and invasion might be potential markers for monitoring malignant phenomena. Franz M. et al reported that tenascin-C(L) and laminin-5 γ2 chain proteins have key functions in the cascade of invasion and metastasis of OSCC.[67]. Their expressions at both the messenger RNA and protein levels are significantly high [68-70].

Chuang et al [71] reported that high podoplanin expression was statistically significantly correlated with clinical nodal metastasis. Some cytoplasmic proteins which regulate oncogenic mechanisms may be potential targets for early tumor detection or tumor recurrence. The expression of serine protease inhibitor, clade B, member 1(SERPINB1) was significantly higher in oral cancer cells with high motility, and the overexpression of SERPINB1 in invasive OSCC was clinicopathologically confirmed [72]. Overexpression of PIN1, a prolyl isomerase which regulates phosphorylation of Ser/Thr- Pro motifs, was associated with progression of OSCC [73]. Overexpression of hypoxia-inducible factor-1α (HIF-1α), which reflects the presence of hypoxia, was correlated with poor survival and tumor progression in patients with OSCC [74-77]. In addition, messenger RNA expression of annexin A1(ANXA1) was found in the peripheral blood of patients with OSCC. A proteomics approach demonstrated downregulation of ANXA1 in OSCC-derived cell lines. More interestingly, the evidence of nuclear localization of the ANXA1 protein from the cytoplasm to nuclei was associated with poor survival of patients with OSCC [78]. Marked downregulation of plasma membranous ANXA1 was correlated with the poorly differentiated status of OSCC cells [79,80]. Monitoring of the nuclear translocation of NXA1can be a potential marker for a diagnosis and prognosis of OSCC. The receptor activator of nuclear factor-κB (RANK) and its ligand (RANKL), which are involved in osteoclastogenesis leading to bony destruction, were potentially associated with bony invasion in patients with OSCC [81].

Chiou et al. [82] found that enriched oral cancer stem-like cells highly expressed stem/progenitor cell markers such as Oct-4, Nanog, CD117, nestin, CD133 and ABCG2, and positive correlations of Oct-4, Nanog and CD133 expressions with the tumor stage were detected. The presence of typical cells may be correlated with tumor progression.

The prevalence of OSCC in the Asian countries is highly associated with betel quid chewing and smoking. The genetic susceptibility to such environmental carcinogens and the resulting altered molecular expressions might be potential markers for a diagnosis and prognosis of OSCC [83]. Studies in patients with OSCC associated with the use of betel nut and/or tobacco showed significantly altered expressions of genes and proteins such as Her-2, highmolecular-weight microtubule-associated protein 2 (hmw-MAP2), and increased expressions of matrix metalloproteinase (MMP)-1 and MMP-8, and increased activities of Src family kinases in invasive tumor fonts86 and in smoking/betel-using patients associated with somatic mitochondrial DNA mutations [84-88].

In addition, chromosomal mutations as nuclear factor-κB promoter, deletion of chromosomes 4p and 9q associated with poor outcomes of betel-using patients with OSCC, and aberrant copy numbers of cyclin D1 (CCND1) and/or cortactin (CTTN) on chromosome 11q13 correlated to arecaassociated OSCC were also reported. Combinational polymorphisms of DNA repair genes of XRCC1, XRCC2, XRCC3, and XRCC4, and the XRCC4 intron 3 delete genotype were highly associated with people who have the betel nut chewing habit and are susceptible to OSCC; in addition, the polymorphism in the promoter of cyclooxygenase-2-1195A/A contributed to the development of betel-related OSCC [89-93].

## Conclusions

The increasing incidence of SCC of the oral cavity in the younger population, combined with an estimated failure to improve survival rates and the evidence that traditional risk factors may not be responsible for a proportion of oral cancer cases in the young, demonstrate the importance of a better understanding of oral cancer epidemiology.

From this review it is clear that contrasting evidence exists in literature as far as the status of alcohol and tobacco as risk factors for oral carcinoma in young adults are concerned. Many authors [22] have reported that risk factors of smoking and drinking are considered significant aetiological agents in older patients and they are present to varying degrees in younger people.

Genetic studies by Tabor MP et al [94] have indicated that patients diagnosed with SCC at a young age may exhibit predispositions to genetic instability.

This literature review has demonstrated that there is a paucity of research examining risk factors other than alcohol and tobacco. This is probably due to the practicalities of asking patients about a plethora of possible risk factors at the time of diagnosis. Despite the fact of an absence of traditional factors in a significant proportion of younger patients and the relatively short time span for these behaviours, it is now important to examine the other potential risk factors, such as environmental carcinogens, stress, previous viral infections, and familial episodes of cancer. Even if these agents are proved to be unlikely risk factors in this population, data collation is important in order to narrow the possibilities. The factors underlying the disease in this sub-group with no obvious risk factors will be difficult to account for unless a thorough examination of all the possible causes is undertaken.

One comment on the studies reviewed is the arbitrary age cut off points used for inclusion into comparison studies of 'young people'. Not only do the age criteria for young people vary between studies, they also lack epidemiological characterization and this fact may be responsible for blurring the boundaries between cancers arising as a result of carcinogenic exposure to traditional risk factors and the ones arising due to a genetic predisposition or an acquired immuno-deficiency.

Despite the attainments already achieved concerning OSCC diagnosis and therapy, mortality and morbidity rates are still exceedingly high, challenging the available methods of prognosis assessment and encouraging the search for new and better markers, namely, molecular markers that relate comprehensively with known alterations of tumor progression.

The immense diversity found in the field of clinical oncology must be considered from two main perspectives: the biologic distinctiveness of each patient and the biologic distinctiveness of each malignancy. In practical terms, the factors with greater consensual influence on disease outcome include disease staging, extracapsular spread, tumor thickness and resection margin free of disease. In the future, better results in clinical oncology appear to rely on improved understanding of tumor molecular biology.

## Conflict of interest

The authors declare that they have no competing interests.

## Authors' contributions

AZ was involved in the conception and wrote the manuscript. GK, PK and AP were involved in the acquisition of the data. JK, NK and VK was involved in the initiation of the report and made a substantial intellectual contribution to the conception and interpretation of the data. All authors read and approved the final manuscript.
